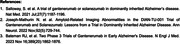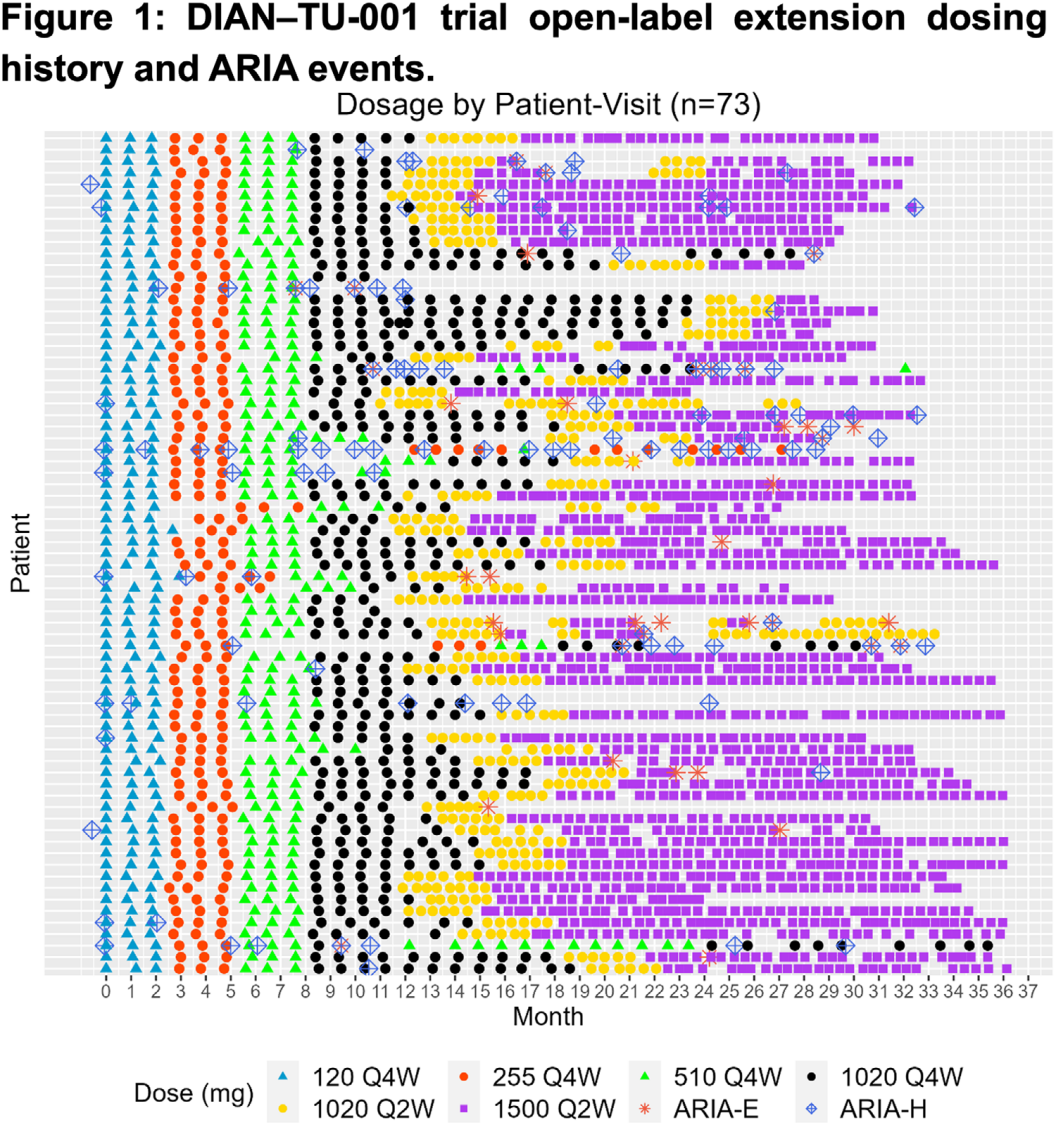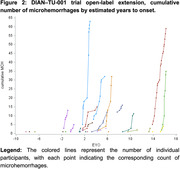# Safety of higher doses of gantenerumab in the open‐label extension of the DIAN–TU‐001 trial

**DOI:** 10.1002/alz.094830

**Published:** 2025-01-09

**Authors:** Jorge J. Llibre‐Guerra, Yan Li, Daniel Yen, Nelly Joseph‐Mathurin, Guoqiao Wang, Andrew J. Aschenbrenner, Chengjie Xiong, Jason J. Hassenstab, Brian A. Gordon, Tammie L.S. Benzinger, Richard J. Perrin, Carsten Hofmann, Jakub Wojtowicz, Alireza Atri, Eric McDade, Randall J. Bateman, David B. Clifford, Dominantly Inherited Alzheimer Network Trial‐Unit.

**Affiliations:** ^1^ Washington University in St. Louis, School of Medicine, St. Louis, MO USA; ^2^ Washington University in St. Louis, St. Louis, MO USA; ^3^ Washington University School of Medicine in St. Louis, St. Louis, MO USA; ^4^ F. Hoffmann‐La Roche Ltd., Pharma Development Safety Risk Management, Basel Switzerland; ^5^ Banner Health, Phoenix, AZ USA

## Abstract

**Background:**

The double‐blind (DB) period of the DIAN–TU‐001 phase 3 trial with gantenerumab provided evidence of significant but incomplete reduction of amyloid plaques, cerebrospinal fluid total tau, and phospho‐tau181 in dominantly inherited Alzheimer’s disease (DIAD).^1^Subsequently, eligible participants transitioned to an open‐label extension (OLE) period using higher doses of gantenerumab (1500mg SC‐administered every two weeks [q2w]).

**Method:**

73 DIAD participants entered the OLE period. All participants titrated gantenerumab doses and received at least 3 doses at 120, 255, 510, and 1020mg‐q4wk. Subsequently, titration continued with 1020mg‐q2w for 6 doses and then to 1500mg‐q2w. During the OLE period, 62/73 (84.9%) participants completed titration to the higher doses (1020‐1500mg‐q2w).

**Result:**

Overall, 30.1% (22/73) of the participants experienced ARIA‐E during the OLE (Figure 1); among those, 72.7% (n = 16/22) experienced ARIA‐E at higher doses (1020‐1500mg‐q2w). 2/3 (67%) APOE4 homozygous participants developed ARIA‐E, while 5/19 (26%) and 10/51 (19%) heterozygous or noncarriers developed ARIA‐E. Radiologically, 56.3% of the ARIA‐E episodes at higher doses were multifocal and had a predominant occipital distribution. The largest cross‐sectional diameter of ARIA‐E at initial findings ranged from 3 to 81mm. ARIA‐E lesions improved, generally, while dosing was held with a mean time for ARIA‐E resolution of 59.5 days. ARIA‐E frequency was higher in PSEN1 and APP variants (14/61 (23.0%) and 2/7 (28.6%), respectively) compared to PSEN‐2 variants (0/5). Most of the ARIA‐E cases (16/22) co‐occur with ARIA‐H; however, ARIA‐H also occurred in 14/51 participants who never had ARIA‐E. ARIA‐H risk increases as the disease progresses and is less frequent during asymptomatic phases of the disease and EYO ←10 (Figure 2).

**Conclusion:**

Safety data from the DIAN‐TU‐001 OLE study indicate that tripling the dose of gantenerumab is well tolerated in DIAD populations, with the incidence and severity of adverse events remaining comparable to the DB period^1,2^ and without new/unexpected safety findings. Most ARIA‐E episodes occurred after titration to doses of 1020‐1500mg‐q2w (16 of 22 participants with ARIA‐E in the OLE) and within the initial 3‐4 months of receiving the higher doses. Risks for ARIA‐E in DIAD parallel reported risks for gantenerumab in sporadic AD, including ApoE4 status and number of MCH.^3^